# Dissociable Effects of Executive Load on Perceived Exertion and Emotional Valence during Submaximal Cycling

**DOI:** 10.3390/ijerph17155576

**Published:** 2020-08-02

**Authors:** Vicente Ávila-Gandía, Francisco Alarcón, José C. Perales, F. Javier López-Román, Antonio J. Luque-Rubia, David Cárdenas

**Affiliations:** 1 Department of Exercise Physiology, Catholic University San Antonio, 30107 Murcia, Spain; vavila@ucam.edu (V.Á.-G.); jlroman@ucam.edu (F.J.L.-R.); ajluque@ucam.edu (A.J.L.-R.); 2 Department of General and Specific Didactics, Faculty of Education, University of Alicante, 03690 Alicante, Spain; 3 Mind, Brain, and Behavior Research Center (CIMCYC), Department of Experimental Psychology, University of Granada, 18071 Granada, Spain; jcesar@ugr.es; 4 Biomedical Research Institute of Murcia (IMIB-Arrixaca), 30107 Murcia, Spain; 5 Department of Physical Education and Sport, Faculty of Sport Sciences, University of Granada, 18071 Granada, Spain; dcardena@ugr.es; 6 Sport and Health University Research Institute (iMUDS), University of Granada, 18071 Granada, Spain

**Keywords:** rating of perceived exertion, valence, arousal, emotion, mental workload, executive workload, affect emotion, exercise

## Abstract

Endurance physical exercise is accompanied by subjective perceptions of exertion (reported perceived exertion, RPE), emotional valence, and arousal. These constructs have been hypothesized to serve as the basis for the exerciser to make decisions regarding when to stop, how to regulate pace, and whether or not to exercise again. In dual physical-cognitive tasks, the mental (executive) workload generated by the cognitive task has been shown to influence these perceptions, in ways that could also influence exercise-related decisions. In the present work, we intend to replicate and extend previous findings that manipulating the amount of executive load imposed by a mental task, performed concomitantly with a submaximal cycling session, influenced emotional states but not perceived exertion. Participants (experienced triathletes) were asked to perform a submaximal cycling task in two conditions with different executive demands (a two-back version of the n-back task vs. oddball) but equated in external physical load. Results showed that the higher executive load condition elicited more arousal and less positive valence than the lower load condition. However, both conditions did not differ in RPE. This experimental dissociation suggests that perceived exertion and its emotional correlates are not interchangeable, which opens the possibility that they could play different roles in exercise-related decision-making.

## 1. Introduction

### 1.1. Effects of Mental Demand on Endurance Performance and Related Variables

Scientific interest on the combined influences of physical and mental demands on performance in physical tasks, and ensuing decision making, has increased in the last years [1,2,3]. Although a number of studies have explored the impact of mental demands on strategic or precision-related aspects of sports performance [4,5], our focus here will be on endurance tasks. In this narrower sense, (a) several studies have reported lengthy and cognitively taxing tasks to have a deleterious effect on subsequent endurance performance, and ratings of perceived exertion (RPE) [6,7]. In contrast, (b) others have tested the effects of mental load on objective and subjective exercise-related variables during a dual physical-mental task (i.e., in tasks in which mental load is concomitant to physical effort) [3,8].

Predictions from studies of the first type, related to the influence of mental fatigue on subsequent physical endurance performance, rely on the assumption that challenging cognitive tasks generate mental fatigue, and directly or indirectly deplete cognitive resources necessary to sustain effort [9,10]. First, accumulated mental fatigue is expected to increase perceived exertion during subsequent physical effort. Second, volitional exhaustion and effort regulation in self-paced endurance exercise are hypothesized to depend on perceived exertion [6,11,12]. Consequently, mental fatigue induced by mental tasks is predicted to reduce performance, measured, for example, as time-to-exhaustion or distance covered in a treadmill and cycle ergometer endurance tests.

Regarding these types of studies, however, the available evidence is still mixed. Some narrative reviews [13,14], and a recent meta-analysis [15] seem to support the above-mentioned prediction. However, another recent review [16] found that about 50% of the relevant studies reported no significant effects of mental fatigue on endurance performance variables. A second recent meta-analysis reported a non-significant mean effect across studies, after correcting for publication bias [17]. At the present moment, doubts remain regarding whether the variance of results is attributable to experimental boundary conditions (e.g., the nature of mental fatigue manipulations, type of performance measure), or methodological limitations (e.g., small sample sizes, publication bias).

Studies of the second type, analyzing the effects of mental load on objective and subjective exercise-related variables during a dual physical-mental task (that is, an endurance task performed in combination with a mental task), are probably more representative of real-life competitive and training scenarios (e.g., sports in which decisions are to be made during exertion). However, they are also much more uncommon in the literature [3,4,8,18,19]. In general, these studies failed to show consistent effects of mental load on perceived exertion, or time to exhaustion in endurance tests. This lack of effects could be nonetheless attributed to several factors, including (a) ceiling effects (i.e., the level of exertion is too high for effects of mental demands to be detectable) and (b) distraction/dissociation (i.e., mental tasks detracts attention from exertion perception). In the case of results regarding subjective scales (e.g., RPE, pain and feeling scales) inconsistencies in results could also be partly explained by (c) the fact that different studies use different scales for similar constructs, and/or participants interpret such scales in slightly different ways, depending on whether they are asked to make their ratings in only one or several, contrasting scales [20].

### 1.2. Dissociation between Perceived Exertion and Emotional Dimensions in Dual Cognitive-Physical Tasks

In our opinion, however, the importance of a second result from these studies has not been sufficiently emphasized. In both Vera et al. [2], and Cárdenas et al. [8], RPE was not influenced by the presence/absence of a concomitant mental task, but emotional correlates were (particularly, emotional valence, as measured by the valence subscale of the Self-Assessment Manikin (SAM) [21]). In other words, the aversive-appetitive valuation of the dual task was dissociated from the subjective feeling of effort.

Such an effect is important on two fronts. On the one hand, it somewhat complicates the explanation of how subjective perception and valuation of effort determines pacing behavior and endurance performance (see, for example, [22]). Seemingly, there is not a single internal construct on which effort regulation relies. On the other hand, it connects research on mental load effects with the large available literature on the rewarding or aversive properties of exercising, and how these determine motivation to exercise and long-term adherence [23,24]. That literature has listed many factors that influence the degree to which exercise is experienced as more or less enjoyable and arousing, including task-related factors (intensity, duration, temperature) and exerciser-related ones (self-efficacy, hydration, fitness level, experience, weight status) [25,26]. Our results added yet another, and practically relevant, variable to this list.

### 1.3. Rationale and Aims of the Study

The present study attempts to conceptually replicate and extend the results by Vera et al. [3]. First, it explores the effects of executive load on perceived exertion and emotional dynamics during a submaximal effort physical task. In that study, participants underwent two dual physical-mental tasks with matched physical demands but different executive demands (a n-back vs. an oddball task). Participants were asked to cycle at 60% of their heart rate reserve (HRR) and a frequency between 50 and 70 rpm during 45 min. Results showed that increasing executive load of the mental task led to a less positive emotional response to exercise and to a higher level of arousal. At the same time, perceived exertion remained unaffected by the executive load, and was only associated with the accumulation of time in the task (and so, plausibly, with physical fatigue) within each session. These results align with DiDomenico and Nussbaum [18], and Mehta and Agnews [19], who failed to find any effect of mental workload on RPE during moderately demanding dual tasks.

Second, the present study also intends to extend previous results in two directions. On the one hand, recent studies show that sports expertise and fitness level modulate the impact of mental load manipulations on physiological indices (heart-rate variability, HRV) [27], so the question arises of whether results obtained with participants in the intermediate range of fitness are generalizable to sportspeople in the high fitness range (experienced triathletes). On the other hand, to date, executive load has been manipulated across conditions matched in terms of physical load. However, physical load matching was ensured by using only external load measures (speed, distance, power output). Here, we also measured indices of internal physical load during submaximal cycling, and controlled for them when necessary, in order to interpret the observed differences in subjective ratings across mental load conditions.

In summary, we hypothesized executive load to reduce task-triggered valence ratings (i.e., to make them less positive), to slightly increase arousal, and to leave RPE scores unaffected, replicating the dissociation found in our previous reports. As some studies have also shown that manipulations of mental demand across conditions matched in external physical demands do not significantly influence physiological parameters like heart rate or blood lactate concentration [28], we do not expect differences in internal physical load indices across conditions.

## 2. Materials and Methods

### 2.1. Participants

Twenty-five triathletes volunteered to participate in the study (mean ± SD age: 29.6 ± 5.8 years; training experience: 4.4 ± 4.8 years; sessions/week: 5.7 ± 0.8; minutes/session: 135.2 ± 28.7; relative oxygen consumption: 56.0 ± 7.2 mL/min/kg; maximum cycling power: 355.6 ± 46.0). We screened participants to meet the following inclusion criteria: (1) triathletes between 20 and 40 years old; (2) with a training frequency of 5 or more sessions per week, comprising at least 2 cycling sessions. Exclusion criteria were (1) serious current or past clinical pathology; (2) serious mental illness (like psychosis or major depression); (3) presence of any absolute or relative contraindication symptom during the initial physical assessment, according to the American College of Sports Medicine [29]. Sample size was based on availability, so no a-priori power analysis was feasible. However, for significant effects of interest, a conservative observed power analysis by simulation was performed using the simr package in R [30].

Participants were informed of their right to quit the study at any time, without the need to provide any reason. Participants gave written consent before the commencement of the study. The study protocol and informed consent were approved by the Ethics Committee of the Catholic University of Murcia (UCAM; ref. 18072014) and were in agreement with the Declaration of Helsinki.

### 2.2. Experimental Design

**Figure 1:**
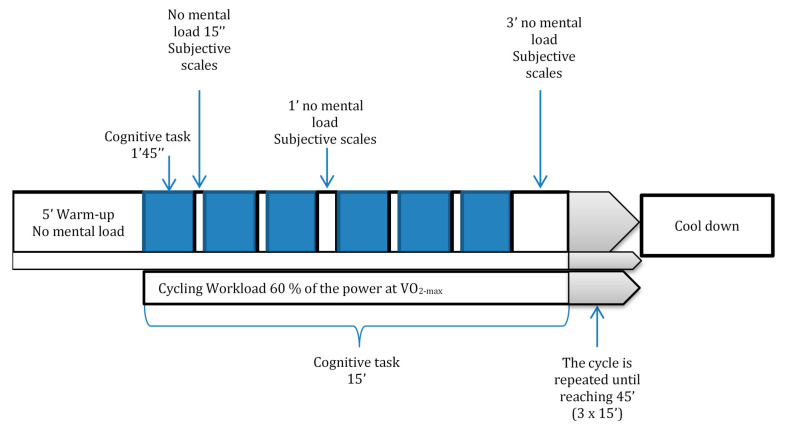
Graphical description of one of the experimental sessions.

In order to assess the effect of mental load on subjective correlates of physical effort, the participants performed two constant-intensity cycling tasks, closely matched in external load, and diverging in the programmed executive load level of the concomitant cognitive task. The executive load was thus experimentally manipulated following a within-participant design (high executive load: n-back vs. low executive load: oddball task). Main dependent variables—emotional valence, arousal, and perceived exertion (RPE)—were assessed at 18 time points in each condition, following the general procedure described below, and depicted in Figure 1. Perceived exertion was evaluated with the Borg RPE scale [31], and valence and arousal with the respective subscales of the Self-Assessment Manikin (SAM) [21]. Heart rate (HR) and oxygen consumption were continuously monitored during the whole of both sessions.

### 2.3. Apparatus and Tasks

#### 2.3.1. Pre-Experimental and Experimental Physical Effort Tasks

In a first pre-experimental session participants performed a maximal incremental cycling test aimed at estimating the maximum level of oxygen consumption (VO_2max_ at exhaustion). This datum was later used to calculate the individual physical workload to be applied in the two experimental sessions. During this test (and the two subsequent experimental sessions), a physician was always present, and a defibrillating device was available in the room.

This maximum physical effort task consisted of a 3 min warm-up at a self-paced intensity, followed by an incremental exercise test to exhaustion (IETE; initial load: 50 watts (W), with a 35 W step increment every minute), on an electronically braked cycle ergometer (Cyclus2, RBM elektronik-automation GmbH, Leipzig, Germany), at a self-selected cadence between 60 and 100 revolutions per minute (RPM), on a fixed gear selected at the beginning of the test. Volunteers were verbally encouraged by the staff to exert maximal effort. Exhaustion was deemed to occur when the subject decided to stop, when pedal cadence dropped 20 RPM below the minimum cadence established (i.e., 40 RPM), or when power output could not be further maintained despite encouragement. This circumstance was interpreted as confirmation of volitional exhaustion.

Heart rate was monitored continuously using an electrocardiograph (included in the Oxycon equipment), and oxygen consumption (VO_2_) was also continuously collected using an automated breath-by-breath system (Jaeger Oxycon ProTM, CareFusion, Höchberg, Germany), recalibrated before each test. All measures were analyzed using the software LABManager 5.3.0.4 (VIASYS Healthcare GmbH, Höchberg, Germany), and were stored in a personal computer for later treatment and analysis. The maximal effort was established following Casajús et al. [32] criteria, namely a plateau of VO_2_, respiratory quotient (RQ) above 1.10, and HR above 95% of the theoretical maximum HR.

In the experimental sessions, a constant intensity cycling task (a square-wave endurance exercise task, SWEET; [33]) was performed using the same cycle ergometer. Participants were instructed to complete a self-paced 5 min warm-up, without reaching initial load, followed by 45 min of SWEET with an individual load in watts, corresponding to 60% of VO_2max_ as calculated in the pre-experimental test, with a pedaling cadence between 60 and 100 RPM. HR was continuously monitored using a pulsometer (Polar RS800CX, Polar Electro Oy, Kempele, Finland) to check that athletes remained under ventilatory threshold 2 (VT2) at the given intensity. Participants also followed the hydration protocol recommended by Palacios et al. [34], namely triathletes drank 150 mL of water every 15 min (after 15, 30, and 45 min).

#### 2.3.2. Mental Workload Tasks

The cognitive task corresponding to each experimental condition (n-back or oddball) was run simultaneously to the submaximal physical effort task described above and started upon reaching the 60% of VO_2max_. The differences in executive load in different versions of the n-back task (e.g., 1-back, 2-back, 3-back) can be operationalized as resulting from the number of items for comparison (digits) the participant is asked to maintain and operate within working memory. The oddball task is actually the 0-back version of the n-back task. That does not mean that the oddball task generates no load, but that the two task types are equated in all potential sources of load except working memory (i.e., executive) load. We chose these mental tasks based on previous studies, revealing that the current (2-back) version of the n-back condition induces higher levels of executive load than the oddball condition [3,35].

The mental workload task (a 2-digit load version of the n-back task; [36]) consisted of a series of digits (1, 2 or 3) presented randomly, one at a time and at a rate of one digit every 2500 ms (each digit was presented for 1000 ms). In each trial, the participants were asked if the digit currently on the screen was the same as the one presented two positions earlier, and they were requested to press a button each time a match was observed (participants did nothing if there was no match). This task thus requires keeping the last two digits in working memory (working memory load), comparing every new digit with the earliest of them (checking), incorporating the new item, and discarding the earliest one for further comparisons (updating).

The oddball condition was designed to be perceptually identical to the n-back task. Before the task started, a randomly selected digit (1, 2 or 3) was presented on the screen, and the participant was instructed to press the button every time that digit appeared on the screen during the ongoing session, and to withhold the response for the other two digits. This task imposes little working memory (i.e., executive) load, but uses the same stimuli as the n-back task, requires vigilance during the whole session, and the same rate of response (on average, one response/3 trials) [35].

Stimuli of the mental load tasks were displayed on a 1920 × 1080 LCD monitor, situated 3 m in front of the participant in order to avoid sustained accommodation (~0 D), while the participant was cycling. Visual stimuli subtended 8.83 min of arc, which corresponds to 0.11 visual acuity, and were thus clearly visible for any participant of this study. The illuminance of the room was quantified with an Illuminance Meter T-10 (Konica Minolta Inc., Tokyo, Japan), and kept constant during the entire experiment (mean ± SD; 249.04 ± 6.47 lux). While cycling, participants held a clicker on their dominant hand to make the responses required for the cognitive task, and a distinctive sound was used as feedback for each response.

### 2.4. Measurements

#### 2.4.1. Rate of Perceived Exertion (RPE)

The CR10 RPE scale [31] allows athletes to subjectively estimate the intensity of the physical effort they are exerting at a certain moment in a numerical cardboard scale ranging from 0 (“nothing at all”) to 10 (“extremely strong”). This scale is useful to screen, prescript, and regulate exercise intensity and assess training load [37] with high convergent validity (r = 0.80−0.90) with physiological measurements [38].

#### 2.4.2. Self-Assessment Manikin (SAM)

The Self-Assessment Manikin (SAM) [21] was used to record the emotional responses evoked by the task. For our purposes, only valence (pleasure-displeasure) and arousal (activation) dimensions were used. This scale was applied at the same points as the RPE scale. This questionnaire is a pictorial, nonverbal assessment tool in which each dimension is represented by 5 icons, displayed over a horizontal line (with an intermediate value between each face) with a total of 9 possible answers, with 1 corresponding to the lowest arousal (the most negative valence) and 9 as the highest arousal (the most positive valence). Subjects were asked to point the finger provided below each of the emotion figures [39].

#### 2.4.3. Cognitive Performance on the Mental Load Tasks

Regardless of the task (n-back or oddball), each trial-wise response qualified as a hit (correct click), a false alarm (or commission error, incorrect click), a correct rejection (correctly non-clicking), or a miss (omission error, incorrectly non-clicking). As a manipulation check of the mental load manipulation, and to ensure the involvement in the cognitive task, we computed the number of hits, misses, false alarms, and correct rejections in each block. Subsequently, we calculated the hit rate *h* (hits/total number of go trials) and the false alarm rate *f* (false alarms/total number of no-go trials) for the entire mental task. Participants performed a total number of 18 blocks. A composite measure of discriminability for each block was computed from *h* and *f* as A’, following Stanislaw and Todorov’s recommendations [40]. The larger A’, the better performance in the task, with A’ = 0.5 meaning performance at chance. Results regarding analyses of performance in the cognitive tasks are reported in Appendix A.

#### 2.4.4. Nutritional Assessment

Dietary habits of the participants were recorded using a validated food questionnaire [41]. Subjects did not change their usual diet during the study period. For the day before, and the same day of any performance test, volunteers had to comply with a previously detailed diet designed by a nutritionist. This included refraining from ingesting caffeine, or any other ergogenic aids or drugs which could affect performance measures. Volunteers were instructed to refrain from performing mentally-demanding tasks in the 24 h before testing.

### 2.5. Procedure

#### 2.5.1. First (Pre-Experimental) Session

The pre-experimental session was scheduled 7 days in advance of the experimental session. Upon consent, participants received instructions and practice with the two versions of the mental load task (n-back and oddball), were familiarized with the subjective scales following Borg [31], and SAM [21] indications, and performed the maximum effort test to exhaustion (IETE), as described earlier.

#### 2.5.2. Second and Third (Experimental) Sessions

The two experimental sessions were conducted at the same time of the day and on the same day of the week, with a one-week separation between them. This consisted of two dual (physical-cognitive) tasks. The two mental load conditions were counterbalanced across participants. RPE and SAM records were collected 18 times at predetermined intervals (approximately once every 2 min; see Figure 1) during the 45 min of continuous (and concomitant) cycling. The order of subjective assessments in each measurement point was changed between participants and measurement points, to control for carryover effects.

The mental workload task (45 min) was divided into three sets of six 105-s blocks, each of which was followed by a mental rest period. The durations of these recesses are shown in Figure 1. The physical task was not interrupted during recesses. During the long breaks subjects were allowed to drink 100 mL of water (according to sports hydration protocol by Palacios et al. [34]). All participants performed 18 blocks so that this was, therefore, the number of blocks considered to check cognitive involvement and performance, and the number of measurement points for subjective scales (RPE, valence, and arousal).

## 3. Results

The code and data for the analyses reported here are available at the Open Science Framework (OSF): https://osf.io/mr8bq/?view_only=5ec6bbde578c4545a72d7303785f0204.

### 3.1. Indices of Internal Physical Load

As noted earlier, the experiment was designed to maintain external physical load constant across the two mental load conditions, which was ensured by setting the cycle ergometer to automatically regulate resistance to keep power output constant. Complementarily, HR, VO_2rel_, and RER indices were used to test whether the two conditions were also matched in terms of internal physical load. Task conditions slightly (and inconsistently) differed in these measures, which calls for the necessity to control for them in further analyses. For the sake of readability these analyses are reported in Appendix A.

### 3.2. Valence, Arousal, and RPE (Joint Analysis)

Valence, arousal, and RPE scores were firstly analyzed together. In order to do so, the three measures were separately standardized, and the sign of valence scores was reversed. Reversing the valence score allowed the three measures to relate with effort in the same direction (arousal and RPE scores are known to correlate positively with effort, whereas non-transformed valence does so negatively) [42]. Given that the aim of the present analysis was to test whether task type (n-back, oddball) exerted different effects on the three subjective scores, ensuring that the three scores are expressed in a common scale and oriented in the same direction facilitates interpretation.

A saturated linear mixed-effects model (LME) was initially built with measure type (valence, arousal, RPE), time in the task (with 18 measuring points, and decomposed in a linear and a quadratic component), and task type (oddball, n-back) as fixed-effects factors, and participant as a random-effects factor. Additionally, and given the differences across task conditions previously observed in internal physical load measures, HR, VO_2rel_, and RER (as well as their interactions with measure, time in the task, and measure × time in the task) were also included in the saturated model. This model with the quadratic and linear components for time (and their interactions) was first tested against a simple one without the quadratic component, and clearly outperformed it. Details of such analysis can be found in the R code accompanying this paper.

This model is conceptually similar to a repeated-measures ANOVA/ANCOVA, with the difference that covariates are allowed to vary within-participant. The effects of theoretical interests in this model are the ones involving task type, as the aim was testing whether the effect of task differed across measures (i.e., the task × measure interaction).

In order to simplify this model, in the first step, the task type × time in task × measure interaction was subtracted from the saturated model (i.e., model 1). As shown in Table 1, subtraction did not hamper model fit, so model 1 was established as a reference model for further comparisons. In the following step, the task × measure interaction was removed from model 1 (i.e., model 2). In this case, the model lost fit. This indicates that the task × measure interaction substantially contributes to variability in the dependent measure. In view of that, the interaction was retained, and model 1 was established as the best-fitting model.

Model 1 was run and used for effect estimation. Estimates and *p*-values for effects in the model are reported in Table 2 (for the sake of simplicity and readability, the effects of covariates and their interactions are not reported). Regression coefficients for time effects in this table do not represent the direction of the effects of time on subjective scales. Arousal and RPE increased, and valence became less positive as the task progressed, but internal physical load also increased in parallel. Consequently, given the relationship between time and internal physical load, statistically removing the effect of internal physical load variables rendered these regression coefficients virtually meaningless. For a more intuitive depiction of the relationship of time and subjective scales, see Table A5. Given that the measure type factor has three levels, its effect was decomposed in two orthogonal contrasts (C1: arousal vs. valence, and C2: arousal + valence vs. RPE). For contrasts, the significance threshold was corrected for the number of contrasts as *p* = 0.05/2 = 0.025.

**Figure 2:**
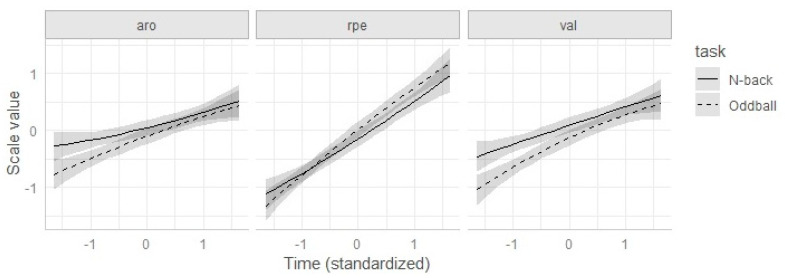
Predicted values from the best-fitting model for subjective scales: arousal (aro), valence (val), and RPE (rpe).

This collection of effects reveals a relationship between time in the task and subjective scores across measure types, a global effect of the task for the three measures together, and different time dynamics across measures and tasks (see Figure 2). However, as noted above, the only effect of theoretical interest is the measure type × task effect. As shown in the table, this effect is restricted to C2, that is, to the interaction between task and the difference of the two emotional scales (valence and arousal) with RPE. More specifically, whereas the n-back task elicited higher arousal and valence scores (indicating more intense arousal and less positive valence), it exerted no detectable effect on RPE. There is, however, no differential effect of task between valence and arousal.

Results regarding the task and task × measure effects were virtually identical when the covariates were removed from the model (see Table A5 and Figure A3).

As noted earlier, the availability of participants did not allow for an a-priori power analysis. We used the lower bound of the observed estimate for the significant C2 × task effect (0.07; see Table 2) for a conservative observed power analysis by simulation (using the *simr* package in R) [30]. This analysis yielded an estimated power between 77.6% and 92.13% for such an effect (95% CI).

### 3.3. Valence, Arousal, and RPE (Segregated Analyses)

Given the differential effect of task type across subjective scales, we carried out separate analyses for the three scales. Three models were built, each with the corresponding scale as the dependent variable. Task and time in the task (including a linear and a quadratic component) were the fixed-effects factor of interest. Internal physical load variables and their interactions with time were included in the model for control purposes. The participant was the only random intercept in the three models.

Effects and their *p*-values are shown in Table 3. Following the interpretation of the joint analysis, task exerted a significant effect on arousal and valence, but not on RPE (actually, the trend of the non-significant effect of task type on RPE was in the opposite direction). The task × time in the task interaction reached significance only for arousal scores.

## 4. Discussion

The main objective of the present study was to replicate previously reported effects [3] of manipulating mental (executive) workload on perceived exertion (RPE), affective valence, and arousal, during a dual physical-mental task, in a sample of well-trained triathletes. At difference with previous studies, we controlled for both externally measured load (power output), and internal physical load measures (oxygen consumption, heart rate, and RER). As expected, valence became less positive, and arousal and RPE increased with time in the task, reproducing the well-known effect of accumulated physical fatigue on RPE and its emotional correlates [43,44]. The two experimental conditions were automatically matched in external physical load (power output), and slightly and inconsistently differed in internal physical load indices. Changes in internal load indices across conditions could be attributed to changes in pedaling pace. As power output was regulated by the cycle ergometer, the only degree of freedom for the participant was cadence. Tentatively, cadence selection could be accounted for by the interference between the goals of the executive and the ones of the physical task [45]. In a recent study, the adoption of a low cadence, corresponding to the energetically optimal cadence, reduced oxygen uptake (VO_2_) during a cycle-run session, compared with the selection of higher cadences (80–90 rpm) [46,47].

The most important result was, however, the finding that the effect of mental task type (i.e., the executive load manipulation) neatly differed across scales. The higher executive load condition (n-back) elicited a less positive exercise-triggered valence, and a more intense arousal state, than the lower load one (oddball), but the two conditions did not differ in RPE. In other words, confirming previous reports, we found a clear dissociation of the effects of executive load on perceived exertion, as measured by the Borg RPE scale, and its emotional correlates, as measured by SAM subscales.

**Figure A2:**
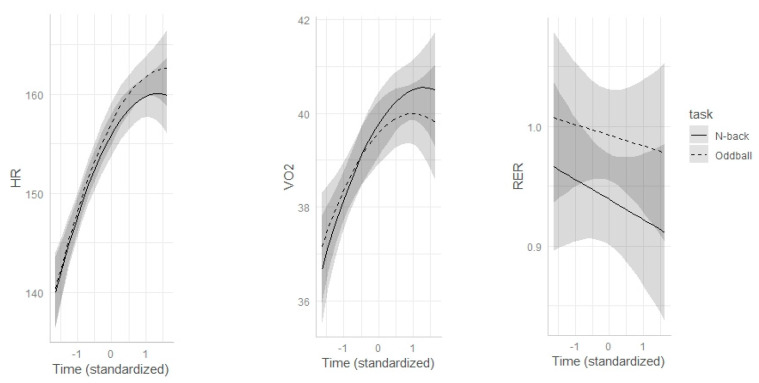
Predicted values from mixed-effects models of internal physical load indices. Left panel: HR as a function of task and time in the task (scaled). Middle panel: VO_2rel_ as a function of task and time in the task (scaled). Right panel: RER as a function of task and time in the task (scaled).

**Figure A3:**
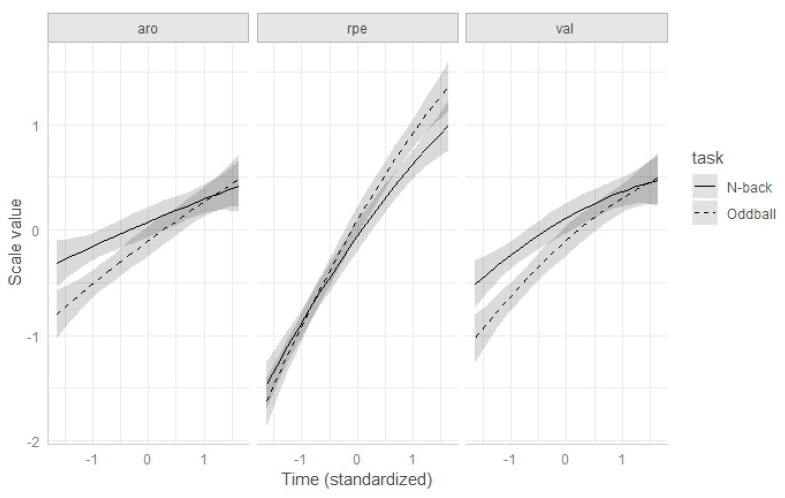
Predicted values from the best-fitting model for subjective scales (arousal, valence, and RPE) with no internal physical load covariates.

Concerning arousal, our results are in accordance with reports that both physical [48] and mental [49] demands increase general cortical activity, which could be the result of higher processing demands during exercise. In accordance with Vera et al. [3], these effects were interactive, as reflected by the significant time (linear) × task type effect in Table 3. As depicted in Figure A2 and Figure A3, the effect of task type was more noticeable in the first part of the task, in which physical fatigue and perceived exertion were lower, but tended to vanish as physical demands increased (i.e., in the last portion of the session).

Regarding valence, a series of works have shown its inverse association with executive workload and complexity of the task [3,8,50]. In other words, the affect state elicited by exercise can turn less positive (or more negative), not only as a consequence of exercise intensity or volume, but also as a consequence of the mental operations necessary to perform such exercise or concomitant to it.

Actually, it has been suggested that negative effects of cognitive and physical load can additively accumulate [51,52], thus increasing total load as experienced by the athlete. Our results, however, yield a more nuanced picture. First, the effects of executive load and time in the task on valence were not additive, but interactive. As it happened with arousal, the effect of executive load on valence was restricted to the first part of the task but became undetectable as the session progressed. This interactive pattern does not seem compatible with the possibility that the high executive load condition could generate more accumulated mental fatigue as the task progresses. If that were the case, the reported effect (the high-load condition elicits less positive valence than the low-load condition) should enlarge with time in task, not the other way around.

Instead, the observed temporal dynamics could be accounted for by cross-task priority dynamics. For example, participants could initially prioritize the physical task, but accrual of errors in the cognitive task would make them redirect their attention to it, which would reduce the number of errors and thus its negative emotional impact [53,54,55]. However, according to this explanation (or the opposite, i.e., the physical task becomes gradually more demanding, detracting attention from the cognitive task and thus reducing its emotional impact), redirecting attention from or to the cognitive task would result in changes in performance in such a cognitive task. These were not observed in the present study.

To date, there is not a straightforward explanation for this interaction. Still, it raises some interesting questions regarding the effect of executive load on global perceptions of exercise enjoyment and future decisions to exercise [56]. In general, pleasantness/unpleasantness judgments for affect-laden activities, and willingness to re-engage in them are sensitive to recency effects [57], namely, they are disproportionally influenced by how such activities end, and not so much by how they evolve. Future research should clarify if the temporal dynamics observed here generalize to different populations, activities, and intensity and duration ranges, and how they affect global pleasantness judgment and decisions to exercise.

Second, and most importantly, the very concept of “total load” is also undermined by the fact that, although executive load influenced valence, it exerted no effect at all on perceived exertion. Any potential effect of executive load on exercise-related decisions (to continue or to discontinue it; to re-engage or not), at least with the type, duration, and intensity of exercise used here, would be mediated exclusively by affective variables, but not by perceived exertion.

In summary, the results presented here are not totally compatible with the idea that mental and physical tasks compete for the volitional resources available in a general pool, as proposed by the ego-depletion hypothesis [58]. They do not fit either with the proposal that displeasure is just a dimension of a perceived exertion [22,59]. It has also been proposed that regulating pace during running or cycling requires the maintenance and updating of exercise-related goals in the working memory [60] so that these physical tasks are by definition also cognitively demanding. These demands, generated by the physical task, would require the involvement of the same control processes involved in the n-back task, generating conflict [61,62], which also has negative affective consequences [63,64]. Testing this hypothesis, however, would require comparing two tasks differing in physical demands, but matched in cognitive demands, in measures of cognitive performance and perceived mental load. Our results show that, during moderate-intensity exercise, increasing executive demands does not contribute to an increased general perception of physical exertion, but they are silent concerning the possible effect of physical demands on perceptions of mental workload.

From the methodological point of view, the soundness of the results presented here is reinforced, on the one hand, by the fact that differential effects of executive load not only emerged in separate scale-by-scale analyses (differences that could be accounted for by the differential sensitivity of the scales or insufficient statistical power), but also as a scale × task type interaction in the joint analysis with the three subjective scales. On the other hand, results did not depend on the above-mentioned differences in internally measured physical load (i.e., remained virtually unaltered regardless of the inclusion or exclusion of such measures as control variables in the analyses). To our knowledge, no previous studies have used a statistical approach that allows controlling for the potential influence of both external and internal physical load, not only between-task, but also within-task.

On the side of limitations, (a) sample size was determined by the availability of participants meeting the inclusion criteria. Sample size (*n* = 24) is however above the average for the field, especially if we take into account that all theoretically relevant manipulations were within-subjects, and LME modeling, as implemented here, is robust even when data points are missing (i.e., if internal physical load recording non-systematically failed for a participant at a certain measurement point). Moreover, (b) data reflect the intrinsic difficulty of strictly controlling for physical load while manipulating other factors (e.g., executive load) in this type of submaximal effort tasks. As long as the task allows for some degree of freedom for the athlete, controlling for the internal physical load will not ensure that external load is also controlled, and vice versa. This calls for the necessity, not only to control for covariates (as done here), but also for replicating the effects of manipulations across slightly different experimental protocols, in order to ensure their generalizability.

## 5. Conclusions

Perceived exertion, and the intensity of emotional aspects of exercise are important because they have been hypothesized to be the main psychological constructs people use to decide whether to continue, discontinue or modulate physical effort during exercise (e.g., “this is too exhausting or too displeasing to continue”), and to later re-engage or not in it. In general, it has been assumed that perceived exertion, arousal, and displeasure change in parallel in response to mental and physical demands. Our results suggest, however, that emotional dimensions of endurance tasks with mental demands (that is, most real-life physical tasks) can vary as a function of variables that do not affect perceived exertion. In other words, increasing executive load during exercise makes the task less pleasing and more arousing, but not more physically effortful. So, a crucial question arises, concerning which one is more influential on exercise-related decision-making. This question opens new lines of investigation with important practical implications.

## Figures and Tables

**Table 1:** Models resulting from hierarchical fitting of valence, arousal, and reported perceived exertion (RPE) scores.

Model	Effects	AIC	df	c^2^	*p*
Saturated	Task, Measure, Time in task (and interactions) *	4845.5	47		
Model 1 **	Saturated minus Task*Measure × Time in task	4843.0	43	5.48	0.241 (M1 > Saturated)
Model 2	Model 1 minus Task*Measure	4869.6	41	30.56	<0.001 (M1 > M2)

* All models also include internal physical load variables and their interactions with time in task and measure. ** Best-fitting model. AIC: Akaike Information Criterion.

**Table 2:** Estimates and significance values for fixed-effects in the joint model of subjective scales.

Predictors	Estimates	CI	*p*
Intercept	−2.92	−3.94–−1.89	**<0.001**
Time (linear)	−41.60	−68.88–−14.33	**0.003**
Time (quadratic)	−45.20	−68.66–−21.74	**<0.001**
Measure (C1)	−0.98	−1.53–−0.43	**<0.001**
Measure (C2)	−0.93	−1.25–−0.62	**<0.001**
Task	−0.13	−0.19–−0.07	**<0.001**
Time (linear)*Measure (C1)	−53.54	−84.59–−22.49	**0.001**
Time (quadratic)*Measure (C1)	−40.45	−67.45–−13.46	**0.003**
Time (linear)*Measure (C2)	2.14	−15.78–20.06	0.815
Time (quadratic)*Measure (C2)	27.33	11.75–42.90	**0.001**
Time (linear)*Task	6.28	3.48–9.09	**<0.001**
Time (quadratic)*Task	−2.30	−5.08–0.47	0.104
Measure (C1)*Task	−0.04	−0.11–0.03	0.316
Measure (C2)*Task	0.11	0.07–0.15	**<0.001**
**Random Effects**			
σ^2^	0.47		
τ_00participant_	0.48		
ICC	0.51		

Note: σ^2^: residual variance, τ_00participant_: individual variability, ICC_participant_: intraclass correlation coefficient. C1 stands for the contrast between arousal and valence, C2 for the contrast between arousal + valence and RPE. Significant *p*-values are marked in bold.

**Table 3:** Estimates and significance values for fixed-effects in the three separate models of each of the subjective scales.

Predictors	Arousal			Valence			RPE		
	Estimates	CI	*p*	Estimates	CI	*p*	Estimates	CI	*p*
Intercept	−1.88	−3.38–−0.37	**0.015**	−2.00	−3.50–−0.50	**0.009**	−4.80	−6.43–−3.17	**<0.001**
Time (linear)	14.22	−9.73–38.17	0.245	−61.22	−85.30–−37.14	**<0.001**	−25.72	−52.64–1.20	0.061
Time (quadratic)	−15.05	−35.66–5.56	0.152	−52.93	−73.65–−32.21	**<0.001**	−10.64	−33.81–12.53	0.368
Task	−0.22	−0.31–−0.13	**<0.001**	−0.27	−0.36–−0.18	**<0.001**	0.10	−0.00–0.20	0.054
Time (linear)*Task	6.12	3.65–8.58	**<0.001**	1.87	−0.61–4.35	0.140	2.83	0.05–5.61	**0.046**
Time (quadratic)*Task	−1.07	−3.51–1.37	0.391	−1.98	−4.44–0.47	0.113	−0.84	−3.59–1.91	0.548
**Random Effects**									
σ^2^	0.36			0.36			0.46		
τ_00participant_	0.63			0.57			0.46		
ICC	0.64			0.61			0.50		

Note: σ^2^: residual variance, τ_00participant_: individual variability, ICC: intraclass correlation coefficient. Significant *p*-values are marked in bold.

**Figure A1:**
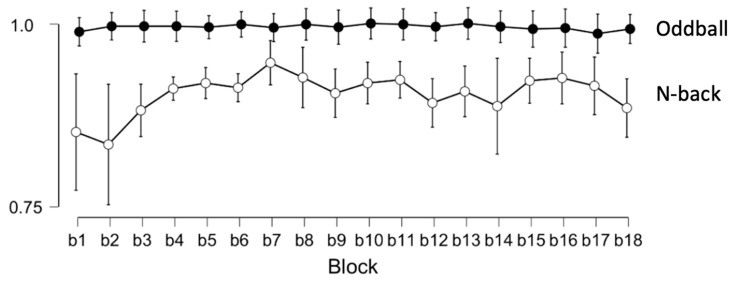
Mean accuracy (A’) in the cognitive task (n-back; oddball) across blocks (bars represent confidence intervals).

## Appendix A. Supplementary Analyses

### Appendix A.1. Analysis of Performance in the Cognitive Task

As detailed in the main text performance (A’) was used to measure accuracy in the cognitive task (with either high or low load, namely the n-back and the oddball versions of the task). This measure ranges from 0 to 1, with 0.5 indicating performance at chance level, and 1 indicating perfect discrimination.

Table A1 displays the results of a block × task within-participants ANOVA, with task representing the version of the task (n-back: high load; oddball: low load), and block the measurement point (with 18 of them throughout the task). As shown in Figure A1, performance approached perfection in the (low load) oddball task, whereas it was lower, although consistently above chance (>0.5) in all blocks of the n-back task. In accordance with the significant block × task interaction, the difference between the two tasks was slightly larger in the first blocks and stabilized after a few blocks (which is probably due to the participants initially familiarizing with the difficult version of the task).

Importantly, these results show, not only that the n-back task was more difficult than the oddball task, actually imposing a higher degree of mental load on participants, but also that performance in the two task was always and consistently above chance, which indirectly shows that participant did not disengage from the task in any part of the cycling session.

**Table A1:** Results of the within-participant 2 (task: n-back, oddball) × 18 (block) ANOVA on performance (A’) in the cognitive task.

	MSE	F	df	p	η²_p_
Task	1.191	25.146	1	**<0.001**	0.626
Residual	0.047		15		
Block	0.006	2.213	17	**0.004**	0.129
Residual	0.003		255		
Task × Block	0.006	2.461	17	**0.001**	0.141
Residual	0.002		255		

Note: Significant *p*-values are marked in bold.

### Appendix A.2. Analysis of Internal Physical Load Indices

HR was analyzed with a linear mixed-effects analysis (LME) with the mental load condition (high, low), and time in the task (with 18 measurement points), as fixed-effects predictors, and participant as a random intercept. Time in the task was scaled and zero-centered to facilitate model convergence, and its effect was decomposed in a linear and a quadratic component, to allow for a curvilinear relationship between time and HR, if necessary. The quadratic component was kept only if IT substantially improved model fit. Model comparison was carried out using the akaike information criterion (AIC), and a χ^2^ test.

The model with the quadratic component (AIC = 4719.3) clearly outperformed the purely linear model (AIC = 4811.6, χ^2^ = 96.31, df = 2, *p* < 0.001), so the more complex model was used to estimate the effects of mental load condition and time in task. Table A2 summarizes the effects of the model. In this and subsequent analyses *p*-values were computed via Wald-statistic approximation; treating t as Wald z.

**Table A2:** Estimates and significance values for fixed-effects in the HR model.

Predictors	Estimates	CI	*p*
Intercept	153.33	148.32–158.33	**<0.001**
Time (linear)	169.06	154.66–183.46	**<0.001**
Time (quadratic)	−54.49	−68.92–−40.07	**<0.001**
Task	1.26	0.49–2.03	**0.001**
Time (linear)*Task	20.06	−0.36–40.49	0.054
Time (quadratic)*Task	3.32	−17.08–23.71	0.750
**Random Effects**			
σ^2^	26.79		
τ_00participant_	160.66		
ICC	0.86		

Note: σ^2^: residual variance, τ_00participant_: individual variability, ICC_participant_: intraclass correlation coefficient. Significant *p*-values are marked in bold.

As expected, HR curvilinearly increased with time in the task. For our purposes, however, the relevant effect is the one of the task, with the sign of the estimate indicating that the predicted HR was 1.26 units faster, on average, in the condition with the oddball task than in the one with the n-back task. The marginally significant time × task interaction revealed that this difference tended to be smaller in the first minutes and larger by the end of the session.

A similar analysis was performed for VO_2rel_. Again, the polynomial model (AIC = 3451.2) clearly outperformed the linear one (AIC = 3468.1, χ^2^ = 20.99, df = 2, *p* < 0.001). Effects in the model are presented in Table A3. In this case, the main effect of task was non-significant, but task interacted with the linear component of the time effect. The sign of the interaction indicated that the VO_2-rel_ measure was slightly higher in the oddball condition by the beginning of the task, but slightly lower by the end of the task (yielding a steeper slope for the time effect in the n-back task).

Finally, for the RER measure, the polynomial model (AIC = 655.12) did not outperform the linear model (AIC = 652.26, χ^2^ = 1.14, df = 2, *p* < 0.565), so the latter was used for estimation (Table A4). In this case, only the effect of the task was significant, with the predicted RER measure showing a globally lower value in the n-back condition.

**Table A3:** Estimates and significance values for fixed-effects in the VO_2rel_ model.

Predictors	Estimates	CI	*p*
Intercept	39.26	37.77–40.76	**<0.001**
Time (linear)	32.60	26.37–38.84	**<0.001**
Time (quadratic)	−10.71	−16.96–−4.46	**0.001**
Task	−0.14	−0.47–0.19	0.408
Time (linear)*Task	−9.74	−18.59–−0.89	**0.031**
Time (quadratic)*Task	0.60	−8.23–9.44	0.894
**Random Effects**			
σ^2^	5.03		
τ_00participant_	14.08		
ICC	0.74		

Note: σ^2^: residual variance, τ_00participant_: individual variability, ICC_participant_: intraclass correlation coefficient. Significant *p*-values are marked in bold.

**Table A4:** Estimates and significance values for fixed-effects in the RER model.

Predictors	Estimates	CI	*p*
Intercept	0.94	0.90–0.98	**<0.001**
Time	−0.02	−0.05–0.02	0.375
Task	0.05	0.00–0.11	**0.049**
Time*Task	0.01	−0.05–0.06	0.772
**Random Effects**			
σ^2^	0.14		
τ_00code_	0.00		
ICC	0.02		

Note: σ^2^: residual variance, τ_00participant_: individual variability, ICC_participant_: intraclass correlation coefficient. Significant *p*-values are marked in bold.

In summary, measures of internal physical load showed different values across mental load conditions, but differences were quantitatively small and did not exactly converge. The three panels in Figure A2 show the predicted HR, VO_2rel_, and RER values from the three models described above. Whereas HR was slightly higher in the oddball condition, oxygen consumption was lower in the oddball condition by the end of the task, and RER was globally lower in the n-back condition. In relation to our aims, however, what these differences show is the necessity to control for them in analyses of valence, arousal, and RPE subjective scores.

### Appendix A.3. Joint Analysis of Arousal, Valence, and RPE without Internal Physical Load Covariates

Table A5 and Figure A3 show the results of analyses equivalent to the ones regarding arousal, valence, and RPE in the main text, excluding internal physical load covariates from the model.

**Table A5:** Estimates for effects in the model for subjective scales without the internal physical load covariates.

Predictors	Estimates	CI	*p*
Intercept	−0.02	−0.30–0.26	0.881
Time (linear)	20.29	18.30–22.27	**<0.001**
Time (quadratic)	−1.80	−3.79–0.19	0.076
Measure (C1)	−0.00	−0.06–0.05	0.866
Measure (C2)	−0.07	−0.10–−0.04	**<0.001**
Task	−0.11	−0.17–−0.04	**0.001**
Time (linear)*Measure (C1)	1.84	0.13–3.55	**0.035**
Time (quadratic)*Measure (C1)	−0.84	−2.56–0.87	0.336
Time (linear)*Measure (C2)	7.78	6.79–8.77	**<0.001**
Time (quadratic)*Measure (C2)	−0.54	−1.53–0.45	0.281
Time (linear)*Task	7.86	5.04–10.68	**<0.001**
Time (quadratic)*Task	−0.51	−3.32–2.31	0.724
Measure (C1)*Task	−0.02	−0.09–0.06	0.644
Measure (C2)*Task	0.11	0.07–0.15	**<0.001**
**Random Effects**			
σ^2^	0.51		
τ_00participant_	0.51		
ICC	0.50		

Note: σ^2^: residual variance, τ_00participant_: individual variability, ICC_participant_: intraclass correlation coefficient. C1 stands for the contrast between arousal and valence, C2 for the contrast between arousal + valence and RPE. Significant *p*-values are marked in bold.

As noted earlier, the availability of participants did not allow for an a-priori power analysis. We used the lower bound of the observed estimate for the significant C2 × task effect (0.07; see Table 2) for a conservative observed power analysis by simulation using the *simr* package in R [30]. This analysis yielded an estimated power between 78.8% and 92.9% for such an effect (95% CI).
